# Age Effects on Old/New Recognition Memory Involving Abstract Figures and Non-words

**DOI:** 10.3389/fnagi.2022.915055

**Published:** 2022-06-20

**Authors:** Monika Toth, Anke Sambeth, Arjan Blokland

**Affiliations:** Department of Neuropsychology and Psychopharmacology, Maastricht University, Maastricht, Netherlands

**Keywords:** discrimination, recognition memory, cognitive aging, abstract figures, non-words

## Abstract

Age-related memory problems posit a growing concern in our society. This study investigated the impact of age and memory strength on recognition memory of pre-experimentally unfamiliar abstract figures and non-words. We applied a three-phase old/new recognition memory paradigm and manipulated memory strength as a function of the Levels of Processing (deep vs. shallow) and repetition. Older adults relative to the young showed impairment in the correct identification of new items. As indicated by the lower discriminability indexes, the older adults also had difficulties discriminating the strongly (drawn/semantically processed) and the weakly (studied) embedded abstract figures but not the non-words. Age-related differences in reaction times were only evident with the abstract figures. Finally, our results revealed that the recognition performance was equally affected by memory strength in both age groups. The current findings agree with previous research on age-related impairment in new item recognition, which can be attributed to misrecollection and decreased sensitivity to novelty in the older adults than the young. The detected age effects on the discriminability of the drawn and studied abstract figures agree with the age-related impairment in the perceptual encoding hypothesis and support the notion related to the need for environmental support to reduce age effects. The lack of age effects with the non-words indicates that age effects on discriminability are stimulus-dependent. The current results support the notion that recognition memory in aging is only impaired under certain conditions and depends on the stimuli used.

## Introduction

Age-related memory problems are among the most prominent complaints of the older adults (Fraundorf et al., 2019). For example, based on oddball tasks, older people have been found to demonstrate decreased sensitivity to novelty (Fandakova et al., 2014), which is likely related to an inability to inhibit irrelevant information (Amenedo and Diaz, 1998; Hasher et al., 1999; Weisz and Czigler, 2006). Although age-related memory deficits in tasks requiring novelty detection, recall, and memory for context are well understood, differences in the processes underlying recognition memory remain unclear.

A recent meta-analysis by Fraundorf et al. (2019) included many studies that used recognition memory paradigms to investigate age effects. In such paradigms, participants have to recognize previously studied items as old correctly and identify previously not seen ones as new (Malmberg, 2008). The recognition/discrimination process generates a cue, which can prompt either a sense of familiarity or novelty (Yonelinas et al., 1998; Yonelinas, 2002). In this context, participants rely on the fidelity of their memory and their preference when making old/new decisions, which can be problematic especially for the older adults. Fraundorf et al. (2019) found that older participants exhibit poorer recognition accuracy, are prone to making more false alarms, and are, thus, more biased toward judging an item “old” even when it is new. Thus, the older adults seem to have the most problems when they have to identify new items. Indeed, previous research has shown that older adults can show decreased sensitivity to novelty (Czigler et al., 2006).

Research has shown that age effects can be reduced when the older adults are encouraged to engage in higher-level encoding (i.e., mnemonic techniques are provided as opposed to rote rehearsal, or when the elderly have to categorize items rather than merely look at them and remember) (Kausler, 1970; Overcast et al., 1975). More engagement leads to deeper levels of processing and stronger memory formation (Craik, 2002; Craik and Rose, 2012). Previous studies have shown that memory strength can effectively be manipulated as a function of repetition (Verde and Rotello, 2007), viewing time (Hirshman, 1995) or depth of processing, the so-called Levels Of Processing (LOP) theory (Gardiner, 1988). The LOP theory predicts that deep (e.g., meaning-extraction, pattern recognition, activation of prior knowledge) and intermediate processing (e.g., phonetics) lead to superior and faster retrieval when compared to shallow processing (e.g., perceptual analyses, rehearsal) (Craik and Lockhart, 1972; Craik and Tulving, 1975; Craik, 2002; Newell and Andrews, 2004).

In agreement with the above, the processing theory account of cognitive aging suggests that the memory problems of the elderly are due to difficulties with commencing mnemonic processing when no experimental instructions are provided and can be improved when such instructions are given (Craik, 2002). Mnemonics, such as verbal (e.g., rhyming), motor (e.g., drawing), or visual (e.g., imagining) techniques, strengthen memory storage and improve subsequent retrieval due to deeper elaborative processing (Paivio and Desrochers, 1981; Jones-Gotman, 1986; Solso, 1995; Hulstijn, 1997). Mnemonics are useful because they involve the allocation of several cognitive domains (Paivio and Desrochers, 1981; Solso, 1995; Hulstijn, 1997) from which the older adults can particularly benefit (Fraundorf et al., 2019).

However, the pictures and words used in most experiments can have an existing representation in memory (Fraundorf et al., 2019). As such, discrimination of such stimuli relies on pre-experimental knowledge, which, especially in the older adults, may lead to source confusion and result in deficits during old/new discrimination. Indeed, Delhaye et al. (2019) reported that the older adults were prone to making more false alarms than the young when they had to learn and recognize pre-experimentally highly familiar word pair recombinations. Thus, the use of pre-experimentally familiar stimuli in recognition tasks may underlie age differences in recognition performance. In contrast, pre-experimentally unfamiliar stimuli, such as abstract figures or non-words, hardly rely on existing semantic knowledge and, therefore, can adequately control for confusion due to prior experience and reveal apparent age effects. For example, Smith et al. (1990), similarly to Harker and Riege (1985), reported significant age differences during the recognition of meaningless abstract but not meaningful, concrete pictures. Another study by Badham and Maylor (2011) compared the discrimination performance of words and non-words in healthy older and young participants using an associative recognition memory paradigm. Age differences in the discrimination of the non-words were comparable to the words, notwithstanding a lack of pre-experimentally existing memory for the non-words.

In the present study, we aimed to examine age effects on the recognition performance as a function of memory strength involving meaningless abstract figures and non-words (Toth et al., 2021a). We chose to include both visual and verbal stimuli in order to examine the effects of aging on recognition performance for these stimulus types differently. It is known that processing of such stimuli can show differential age effects (Koutstaal et al., 2003; Badham and Maylor, 2011). Further, these stimuli are also known to be processed differently in the brain that may differently affected during aging (Kim et al., 2004). To investigate this, we tested older adults and young participants applying a three-phase old/new memory paradigm (Toth et al., 2021a). The stimuli were first familiarized *via* mnemonic encoding to prompt deep processing: the participants had to redraw the abstract figures and mention existing rhyming words for the non-words (semantic processing). Then, the stimuli in the second phase were processed shallowly *via* an instruction to remember as many items as possible. Here, the stimuli from the first phase were shown repeatedly mixed with some new items. Finally, in the third phase, an old/new recognition test was used. Here, stimuli from the first and second phases were shown together with new items. Recognition accuracy and response speed were assessed.

We expected to find age differences concerning the correct recognition of the studied and the new items likely resulting from higher false alarm rates in the older adult than the young group. However, no age effects were anticipated concerning the recognition of the deeply memorized and repeated items relying on strong memories in line with the processing theory account of cognitive aging (Craik and Rose, 2012) and the production deficit hypothesis (Kausler, 1970). Also, the older participants were expected to be slower in processing than the young.

## Materials and Methods

The Ethical Committee of the Faculty of Psychology and Neuroscience of Maastricht University granted ethical approval for this experiment. Each participant received monetary compensation or research participation credit points. On average, the experiment took 1.5 h/test-session (Ethical Approval Code: ECP13_02_2012).

### Participants

An a priori statistical power analysis based on previous data obtained with the current paradigm and using G^*^power 3.1 showed that in order to detect significant effects using an ANOVA involving between and within subject interactions 9 participants were required for each age group, with a effects size of 0.4 based on Cohen's d and power of at least 95% at a significance level of 5% (Faul et al., 2007). The main inclusion criteria were age (young: 18–30 years, older adults 60–80 years) and being fluent in the Dutch language. The older adults were excluded if suffering from any objectified memory impairment, having visual impairments, or neurological/neuropsychiatric disorders. In order to exclude severe cognitive impairment, the Mini Mental State Examination (MMSE) was administered, and volunteers who scored <25 indicating dementia, were excluded (Folstein et al., 1975).

A total of 30 young and healthy older adults were recruited by means of advertising. However, two older adult participants were excluded from the analyses due to a technical failure. Thus, the final sample contains data of 15 young (5 males) with mean age of 23 years, and 13 healthy older adults (8 males) with a mean age of 71 years.

### Procedure

The procedure and the stimuli were the same as applied in another study by our group (Toth et al., 2021a). After signing informed consent, participants were admitted to the study. Before starting the experiment, each participant filled in a demographic questionnaire, including information about sex, age, and handedness. During the test, stimuli were presented *via* a computer screen, and participants had to respond on two keys of a response pad. Recognition accuracies and reaction times were recorded.

**Figure 1:**
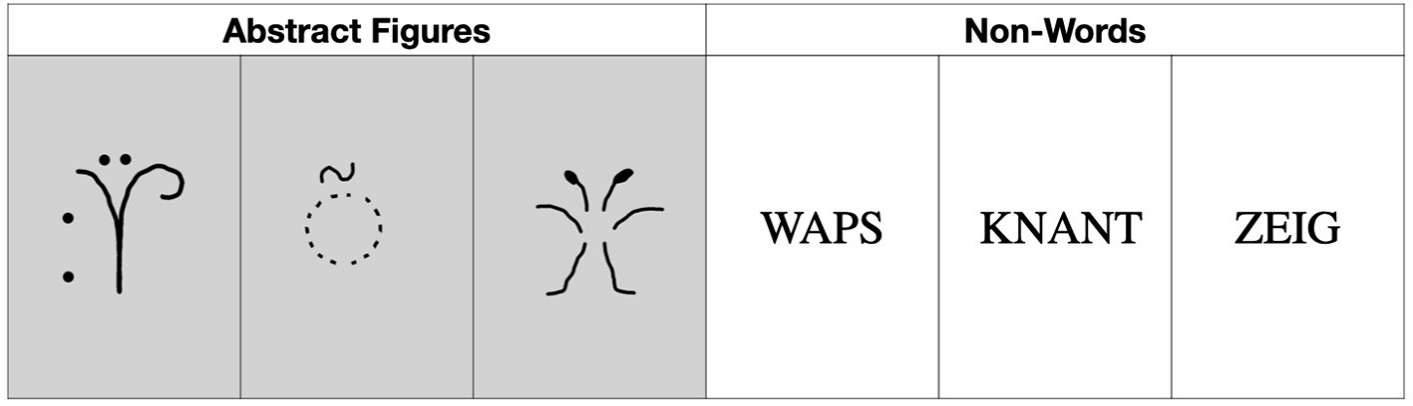
Examples of the stimuli used.

A memory paradigm with abstract figures (i.e., simple line drawings forming pre-experimentally unfamiliar shapes) and non-words (i.e., non-sense letter strings, which could be pronounced) was applied in separate tests. The stimuli were extracted from previous studies and were equally unfamiliar for both age groups (Seidenberg et al., 1994; Glosser et al., 1998; Redoblado et al., 2003). See Figure 1 for an example of the stimuli used.

**Figure 2:**
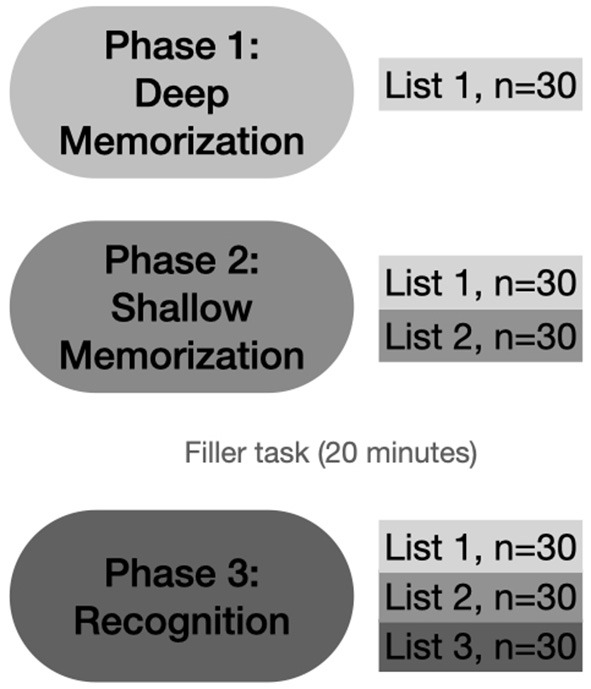
Schematic overview of the experimental design. Phase 1: deep memorization with the pre-experimentally unfamiliar abstract figures and non-words in separate tests using a mnemonic encoding task (redrawing the abstract figures and mentioning rhyming words for the non-words). The 30 stimuli used here form List 1 (drawn/semantically processed stimuli). Phase 2: shallow memorization with the instruction to remember as many stimuli as possible. This phase contained items from List 1 and 30 new ones (List 2, studied stimuli). Phase 3: recognition of the stimuli including List 1, List 2, and 30 new (List 3). N: number of stimuli presented (Toth et al., 2021a).

Every participant performed each test phase first with the abstract figures and then with the non-words to minimize verbalization of the visual stimuli. The experiment consisted of three phases (see Figure 2). In phase 1 (deep memorization leading to “strong” memory formation), participants were familiarized with a series of 30 abstract figures or monosyllabic non-words in separate tests (list 1: L1). Participants were asked to manually redraw the abstract figures on an answer sheet to induce deep LOP. They had to mention existing Dutch rhyme words for each non-word to induce intermediate LOP. Stimuli were presented for 1 s, and the participants were given 14 s to execute the deep encoding task. If they were ready earlier, they could press a button, and 2 s later, the next stimulus appeared.

During phase 2 (shallow memorization leading to “weak” memory), participants were instructed to remember as many stimuli as possible. In this phase, 60 stimuli (abstract figures or non-words) were used: 30 stimuli from L1 were randomly mixed with 30 new ones (L2). All stimuli were shown for 1 s with an inter-stimulus interval (ISI) of 2 s.

During phase 3, participants were asked to decide if they had seen the presented stimulus in the previous series (L1 and L2) or whether the stimulus was new to them (L3: new, *n* = 30). The 90 non-words or abstract figures were presented for a duration of 1 s, or less in the case of faster button press; the ISI was 2.5 s. Participants had to press the corresponding buttons (“old” for L1 and L2, or “new” for L3 stimuli) on a response box as quickly and accurately as possible. The order of the test items was random making sure that no more than 2 consecutive items came from the same phase during the recognition test.

A filler paper-and-pencil task and another non-verbal task were given between phase 2 and 3. The filler task consisted of the localization of number sequences, vertically or horizontally placed within a field of numbers (10 min). The other task consisted of watching a silent cartoon while auditory stimuli were presented (10 min).

### Data Analysis

The analyses was identical to Toth et al. (2021a). Thus, before analysis, all data were evaluated for having normal distribution and homogeneity of variance. Additionally, raw data were checked for outliers. Outlier values were replaced with their regression estimates produced by the missing value analyses module in SPSS (IBM SPSS Statistics for Macintosh, Version 27.0. Armonk, NY: IBM Corporation). Additionally, due to technical issues, 1–2 responses per participant were missing (e.g., the button press was not recorded). In these cases, values were replaced with their regression estimates. Effect sizes are reported based on partial eta-squared (ηp2) data. Furthermore, Mauchly's test of sphericity was applied. In case the assumption of sphericity was violated, a Greenhouse–Geisser correction was used. In all cases, degrees of freedom of assumed sphericity were reported. Levene's test was used to assess the equality of variances. In case the assumption of equal variances was violated, median-based independent samples non-parametric tests were applied. *Post-Hoc* comparisons and simple effects were investigated using paired-samples and independents samples t-tests, applying adjustments for multiple comparisons; the observed p-values were multiplied by the number of comparisons, which was tested against the set significance level of 0.05. Values of unequal variances are reported if the assumption of equal variances was violated.

For the behavioral data, Signal Detection Theory (SDT) was applied to investigate the recognition performance (Stanislaw and Todorow, 1999; Benjamin and Bawa, 2004; Verde and Rotello, 2007; Benjamin et al., 2009). Recognition accuracy was defined as the ability to distinguish the different types of stimuli (drawn/semantically processed, studied and new). Correct responses included an “old” response to the drawn/semantically processed and the studied stimuli, and a “new” response to the new items. Incorrect responses involved a “new” response to the drawn/semantically processed items and the studied stimuli and an “old” response to the new stimuli. See Table 1 for an overview.

**Table 1:** Overview of the different types of responses as a function of stimulus type.

	**Stimulus type**	**Response**
Hit (H)	Drawn or semantically processed/Studied	“Old”
Miss (M)	Drawn or semantically processed/Studied	“New”
Correct Rejection (CR)	New	“New”
False Alarm (FA)	New	“Old”
Hit Rate (HR)	Drawn or semantically processed/Studied	H/(H + M)
Correct Rejection Rate (CRR)	New	CR/(CR + FA)

Given the memory strength manipulation in the current design (deep memorization, shallow memorization, and recognition), the correct response rates, being hit rates (HR) for the drawn/semantically processed, and the studied items and correct rejection rates (CRR) for the new, were used to evaluate the discrimination accuracy. Furthermore, to investigate discriminability, non-parametric A' statistics were computed for the drawn/semantically processed and the studied stimuli using Equations (1) or (2) (see below). A′ is independent of the distribution of the data and varies from 0 to 1, with 0.5 indicating chance performance. Higher values are indicative of improved performance (Snodgrass and Corwin, 1988; Stanislaw and Todorow, 1999).


(1) $$\begin{array}{l} A^{\prime }\;=\;0.5\;+\;\frac{\left(HR\;-\;FAR\right)\left(1\;+\;HR-FAR\right)}{4HR\left(1\;-\;FAR\right)},ifHR\geq FAR \end{array}$$

(2) $$\begin{array}{l} A^{\prime }\;=\;0.5\;-\;\frac{\left(HR\;-\;FAR\right)\left(1\;+\;HR\;-\;FAR\right)}{4HR\left(1\;-\;FAR\right)},ifHR<FAR \end{array}$$

A': discriminability index, HR: hit rate, FAR: false alarm rate.

During recognition, the a priori probabilities of old and new items and the quality of the match between a test item and the memory for studied items can influence the bias parameter (Stanislaw and Todorow, 1999; Huang and Ferreira, 2020). Such a model does not fit the current paradigm due to the memory strength manipulation used and the equivalent proportion and intended comparison of the strong (*n* = 30), weak (*n* = 30), and new items (*n* = 30) (Benjamin and Bawa, 2004). After all, the final proportion of “old” and “new” responses was 2:1. Therefore, we calculated the total amount of “old” (H + FA) and “new” (M + CR) responses given by the participants. This was done to examine whether there was a preference for either the “old” or “new” responses. Results were compared using paired samples *t*-tests with Bonferroni corrections.

RT data of the hits were evaluated, as well. To be able to use parametric tests, RT-s were transformed into |log(1/RT)| to obtain a normal distribution of the data (Osborne, 2002). Moreover, the median RT data are reported as central tendency parameters, together with the corresponding first and third interquartile ranges (Ratcliff, 1993).

Statistical analysis was conducted using SPSS 27.0. A repeated measures analysis of variance (ANOVA) was used to investigate discrimination accuracy scores and RT-s for the different stimuli (drawn/semantically processed, studied, and new) in the different categories as assessed in Phase 3. The within-subject variables were Stimulus Type with three levels for the accuracy scores (drawn/semantically processed, studied, and new) and two levels for the A' scores (drawn/semantically processed and studied) for the abstract figures and non-words analyzed in separate tests. The between subjects variable was Age (young and older adults).

## Results

Although there was an unequal number of old-responses over new-responses (2:1), we found that there was no response bias (see Table 2). The mean signal-detection parameter estimates are displayed in Table 3.

**Table 2:** The overall number of old and new responses during the recognition phase Data represent the means (SEM) of the total “old” and “new” responses and the corresponding % compared to the 90 items/stimulus category (abstract figures and non-words), and the t-statistics for the young and the older adults.

	**Young**		**Older adults**	
**Response type**	**Abstract figures**	**Non-words**	**Abstract figures**	**Non-words**
“Old”	48.40 (2.53) 54%	43.47 (2.18) 48%	49.23 (2.12) 55%	47.85 (3.76) 53%
“New”	41.53 (2.54) 46%	46.40 (2.20) 52%	40.46 (2.02) 45%	42.00 (3.75) 47%
Paired samples
t-test	t_(14)_ = 1.36, *p* > 0.197	t_(14)_ = 0.67, *p* > 0.514	t_(12)_ = 2.12, *p* > 0.550	t_(12)_ = 0.78, *p* > 0.451

**Table 3:** Means (SEMs) of the signal-detection measures concerning the recognition performance with the abstract figures and non-words for the drawn/semantically processed, studied, and new stimuli according to age (young and older adults).

		**Young**		**Older adults**	
**Stimulus type**	**Parameters**	**Abstract figures**	**Non-words**	**Abstract figures**	**Non-words**
Drawn/Semantically processed	HR	0.98 (0.01)	0.80 (0.04)	0.94 (0.12)	0.83 (0.02)
	A'	0.90 (0.10)	0.69 (0.02)	0.76 (0.03) *	0.62. (0.01)
Studied	HR	0.57 (0.06) ^**aa, bb**^	0.50 (0.04) ^**aa, bb**^	0.52 (0.05) ^**aa, bb**^	0.57 (0.05) ^**aa, bb**^
	A'	0.63 (0.03) ^**aa**^	0.56 (0.01) ^**aa**^	0.56 (0.01) *, ^**aa**^	0.55 (0.01) ^**aa**^
New	CRR	0.95 (0.01) ^**aa**^	0.84 (0.02)	0.80 (0.04) *, ^**aa**^	0.70 (0.02) *

HR, hit rate; CRR, correct rejection rate; A': discriminability index. Age effects: ^*^: p <0.05. Stimulus type effects: different from the drawn items: aa p <0.001, different from the new items: bb p <0.001.

### Abstract Figures

**Figure 3:**
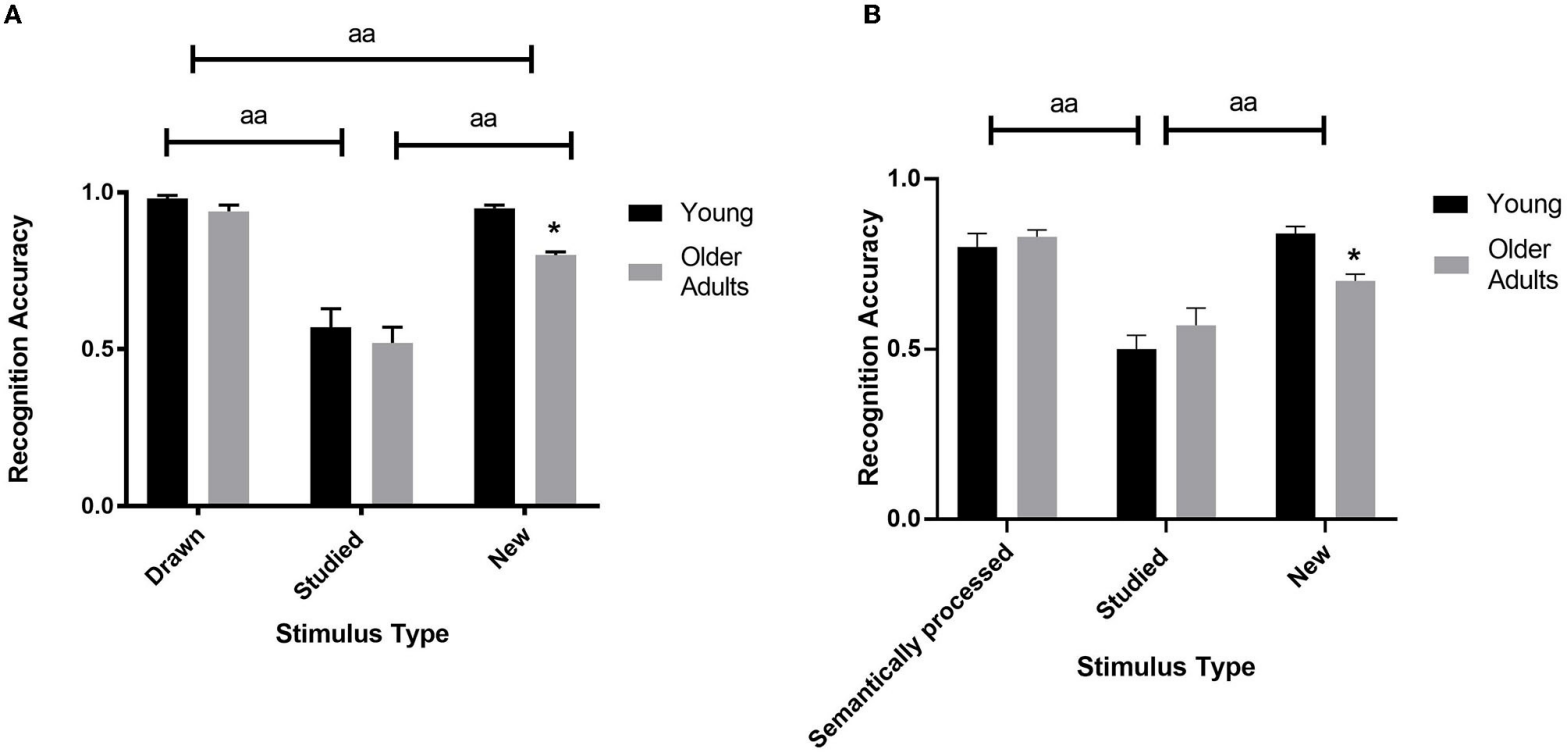
Recognition accuracy performance of the abstract figures **(A)** and the non-words **(B)** according to age (young and older adults) for the hit rates of the drawn/semantically processed and studied items, and the correct rejection rates in repose to the new items. The bars represent the means. Age effects: *: *p* < 0.05. Stimulus type effects: aa: *p* < 0.001.

When analyzing the accuracy performance in the session with the abstract figures the ANOVA revealed a significant age x stimulus type interaction [*F*_(2, 25)_ = 4.00, ηp^2^ = 0.34, *p* < 0.031; see Figure 3A and Table 3]. Levene's test indicated unequal variances for the new abstract figures [*F*_(1, 26)_ = 6.79, *p* < 0.015]. Simple effects analyses revealed that the young compared to the older adults were more accurate in recognizing the new abstract figures [*t*_(12.38)_ = 3.36, *d* = 1.34, *p* < 0.015]. No such differences were found for the drawn [*t*_(26)_= 2.52, *p* > 0.054] and the studied items [*t*_(26)_ = 0.63, *p* > 0.999]. Also, the main effect of age [*F*_(1, 26)_ = 6.13, ηp^2^ = 0.19, *p* < 0.020] and stimulus type were significant [*F*_(2, 52)_ = 76.67, ηp^2^ = 0.75, *p* < 0.001; see Figure 3A and Table 3]. *Post-Hoc* tests of the stimulus type showed that overall recognition accuracy of the drawn stimuli was better compared to the studied (*p* < 0.001) and new (*p* < 0.001). Also, more new stimuli were endorsed correctly compared to the studied (*p* < 0.001).

The analyses performed on the A' scores revealed a significant age x stimulus type interaction [*F*(_1, 26)_ = 4.84, ηp^2^ = 0.16, *p* < 0.037; see Table 3]. Levene's test indicated unequal variances for the drawn [*F*_(1, 26_) = 5.83, *p* < 0.023] and the studied abstract figures [*F*_(1, 26)_ = 4.71, *p* < 0.039]. Simple effects analyses revealed that the young compared to the older adults could discriminate the drawn [*t*_(14.82)_ = 4.77, *d* = 1.91, *p* < 0.001] and the studied abstract figures better [*t*_(22.22)_ = 2.40, *d* = 0.87, *p* < 0.025]. As for the stimulus type effect, the drawn abstract figures resulted in improved discriminability compared to the studied [*F*_(1.26)_ = 203.47, ηp2 = 0.89, *p* < 0.001; see Table 3]. The main effect of age was also significant [*F*_(1.26)_ = 203.47, ηp2 = 0.89, *p* < 0.001].

When analyzing the RT-s, the ANOVA confirmed a significant main effect of age [*F*(_1, 27)_ = 19.56, ηp^2^ = 0.43, *p* < 0.001; see Table 3], with the young participants being faster than the older adults. Another main effect of stimulus type was found [*F*_(2, 25)_ = 85.29, ηp^2^ = 0.87, *p* < 0.001; see Table 3]. *Post-Hoc* tests showed that reactions were faster to the familiarized compared to the studied and compared to the new stimuli (*p* < 0.001) in both age groups. No such difference was found between the studied and new stimuli (*p* > 0.999). Finally, there was no interaction between age and stimulus type [*F*_(2, 52)_ = 1.20, ηp^2^ = 0.09, *p* > 0.319].

### Non-words

The ANOVA analysis of the non-words revealed a significant age x stimulus type interaction [*F*_(2, 25)_ = 4.57, ηp^2^ = 0.27, *p* < 0.020; see Figure 3B and Table 3]. Levene's test indicated unequal variances for the semantically processed non-words [*F*_(1, 26)_ = 6.79, *p* < 0.015]. Simple effects analyses showed that the young compared to the older participants were more accurate in rejecting the new non-words correctly [*t*_(26)_ = 4.42, *d* = 1.68, *p* < 0.003]. No such differences were found for the semantically processed [*t*_(23.21)_ = 0.66, *p* > 0.999] and studied items [*t*_(26)_ = 1.54, *p* > 0.936]. Moreover, the main effect of stimulus type was also statistically meaningful [*F*_(2, 25)_ = 47.83, ηp^2^ = 0.79, *p* < 0.001; see Figure 3B and Table 3]. *Post-Hoc* tests showed that the recognition accuracy of the semantically processed stimuli was better compared to the studied (*p* < 0.001) but not compared to the new items (*p* > 0.340). Also, more new non-words were identified correctly compared to the studied (*p* < 0.001). Age did not yield a significant main effect [*F*_(1, 26)_ = 0.20, ηp^2^ = 0.01, *p* > 0.659].

The analyses of the A' scores revealed a significant age x stimulus type interaction [*F*_(1, 26)_ = 4.89, ηp^2^ = 0.16, *p* < 0.036; see Table 3]. However, simple effects analyses did not reveal any differences for the semantically processed [*t*_(26)_ = 4.77, *d* = 0.87, *p* > 0.060] or the studied non-words [*t*_(26)_ = 4.77, *d* = 0.45, *p* > 0.060]. As for the stimulus type effect, the semantically processed non-words resulted in improved discriminability compared to the studied [*F*_(1.26)_ = 75.76, ηp2 = 0.75, *p* < 0.001; see Table 3]. The main effect of age was not significant [*F*_(1.26)_ = 203.47, ηp2 = 0.89, *p* > 0.070].

The analyses of the RT-s did not yield an age [*F*_(1, 26)_ = 0.62, ηp^2^ = 0.02, *p* > 0.440], or stimulus type effect [*F*_(2, 25)_ = 1.98, ηp^2^ = 0.14, *p* > 0.159]. Finally, the stimulus type x age interaction was statistically also not meaningful [*F*_(2, 25)_ = 0.26, ηp^2^ = 0.02, *p* > 0.773] (see Table 4).

**Table 4:** Median reaction times (middle 50% range; in milliseconds in response to the abstract figures and non-words (the drawn/semantically processed, studied, and new) and their corresponding first and third interquartile ranges for the young and the older adults.

	**Young**		**Older Adults**	
**Stimulus type**	**Abstract figures**	**Non-words**	**Abstract figures**	**Non-words**
Drawn/Semantically processed	661 (613-742)	648 (636-665)	911 ** (781-1029)	659 (627-790)
Studied	794^aa^ (718-1011)	650 (575-719)	1099**, ^**aa**^ (938-1202)	620 (578-651)
New	811^aa^ (734-953)	689 (637-734)	1149 **, ^**aa**^ (1034-1210)	684** (495-782)

Age effects: **p <0.001. Stimulus type effects: different from the drawn/semantically processed: aa p <0.001.

## Discussion

The current study investigated age effects on the recognition performance involving abstract figures and non-words using a three-phase old/new memory paradigm. Our results revealed age-related deficits in new item identification for both the abstract figures and the non-words. Moreover, as indicated by the discriminability indexes (A'), the young were better in discriminating the drawn and studied abstract figures than the older adults. Interestingly, this was not found for the non-words, suggesting a stimulus type dependent age effect. In line with previous research, this was likely due to the older adults making more false alarms (Bowman and Dennis, 2015; Fraundorf et al., 2019). Partly in contrast to our expectations, age-related slowing in reaction times was only evident with the abstract figures but not with the non-words.

In agreement with previous results using the current paradigm, it was found that recognition performance was affected by memory strength in both age groups to a similar extent (Toth et al., 2021a). In contrast, the recognition of the new items was comparable to the semantically processed non-words but was worse for the drawn abstract figures (Toth et al., 2021a,b). Finally, our overall results were in line with our expectation concerning the age-independent memory advantage of deeper LOP and repetition over shallow LOP without repetition (Craik and Lockhart, 1972; Craik and Tulving, 1975; Craik, 2002; Newell and Andrews, 2004). In other words, the deeply processed and repeated items were recognized better than the shallowly encoded ones.

### Age Effects on New Item Recognition

The older adults in the current study could identify the new abstract figures and non-words as “new” less accurately than the young. Accordingly, the older adults produced more false alarms, which explains the age-related deficits in the differentiation of these stimuli. Several accounts can be offered in explanation of these results, such as the misrecollection account of cognitive aging (Dodson et al., 2007) and decreased sensitivity to novelty (Czigler et al., 2006).

First, it is well-documented that during discrimination the older adults can have difficulties with correct identification of new items when the old items are perceived as insufficiently distinct perceptually or conceptually from the new ones (Dodson et al., 2007; Gallo et al., 2007; Fraundorf et al., 2019). As such, new items can prompt an increase in the number of false alarms (Kroll et al., 1996; Gallo et al., 2007). False memories can reliably induce misrecollection, which the older adults are specifically prone to (Dodson et al., 2007).

Second, as a consequence of cognitive aging, sensitivity to novelty may decline (Czigler et al., 2006; Daffner et al., 2006, 2011). For example, it has been shown by Daffner et al. (2006) that cognitively average performing older adults had shorter viewing time in response to the novel stimuli than the older adults with high cognitive performance. The authors attributed this to decreased novelty sensitivity. Similarly, Czigler et al. (2006) also reported decreased sensitivity to visual novelty and overall cognitive slowing in the older adults compared to the young. Therefore, it could be that the results of the current experiment may also reflect an age-related decrease in sensitivity to novelty.

### Age Effects on Old Item Recognition

We anticipated detecting age effects concerning the correct recognition of the studied but not the drawn abstract figures and semantically processed non-words. When looking at the pure hit rates, age effects were not apparent. However, clear age effects were found concerning the discriminability indexes (A') of these stimuli, which involve false alarms. Namely, it was more difficult for the older adults to differentiate the drawn and studied abstract figures. Also, the reaction times of the older adults were slower in response to these stimuli. Interestingly, this was not found for the non-words. Thus, the presented results indicate that the effects of aging seem to be stimulus-dependent.

In contrast to our results with the pre-experimentally unfamiliar stimuli, Ruiz Gallego-Largo et al. (2015) showed that older adults recognized the pre-experimentally familiar pictures better than the young. The opposite was found for the verbal stimuli. A good reason why our results showed the reverse pattern could be attributed to the nature of the stimuli used in the current study. We used pre-experimentally unfamiliar visual and verbal items. This is particularly relevant because prior knowledge, in some situations, can aid the performance of the older adults in recognition memory tests (Umanath and Marsh, 2014; Tinard and Guillaume, 2019), while in other cases, it can hinder their memory performance. For example, Foos and Sarno (1998) showed that when contrasted with young participants, the older adults exhibited an encoding deficit when easily retrievable pre-experimentally familiar information had to be learned and later recognized. The authors argued that this deficit could be due to the older adults feeling overly confident in what they think they know. Such a notion was supported by shorter learning time and less distinctive encoding leading to poorer recognition in the older adults. Taken together, pre-existing differences in semantic memory in different age groups can affect recognition memory. The use of pre-experimentally unfamiliar stimuli may prevent this issue.

Interestingly, both the older adults and the young reacted at a comparable pace to the non-words, whereas we detected a setback in reaction times with the abstract figures. A possible explanation could be that pictures are represented as integrated patterns, whereas verbal stimuli are not (Rajaram, 1996). Moreover, Noldy et al. (1990) suggested that the processing of verbal stimuli is automatic and fast. In contrast, picture processing requires additional allocation of attentional resources, which can slow down reactions. Automated memory processes in healthy aging are preserved and comparable to that in young (Titov and Knight, 1997). Consequentially, this can explain why the processing speed of the abstract figures did show age effects, and that of the non-words did not. Since cognitive slowing was found to be differential depending on the pre-experimentally unfamiliar stimulus being visual or verbal, it seems plausible that age differences do not stem from a single global deficit (Benjamin, 2010; Fraundorf et al., 2019) but are stimulus-dependent. As such, our findings are difficult to reconcile with the notion that the older adults' longer reaction times could be attributed to an overall motor slowing (Woods et al., 2015) or the older adults aiming to minimize errors at the cost of being slower (Salthouse, 1996). Instead, it seems more likely that the slower reactions seen in the visual as opposed to the verbal domain are due to age-related changes in the cognitive ability to process such materials (Glass, 2007; Bisiacchi et al., 2008).

Finally, it is essential to mention that the hit rates in the session with the abstract figures were close to perfect. It has been proposed that ceiling effects for young participants (i.g., overly easy tasks) can mask age effects in recognition memory paradigms (Uttl et al., 2007). However, it seems unlikely that the current paradigm was not sufficiently challenging for the young. Namely, the stimuli were pre-experimentally unfamiliar, the ISI was relatively short, and the participants had to recognize 90 items. Also, the studied items' hit rates were low, and the miss rates were high, indicating that the task was likely difficult for the young. As such, our findings corroborate those of Danckert and Craik (2013), who attempted to control for ceiling effects in relation to age differences in recognition memory. They also used a deep and a shallow encoding task. In addition, they controlled for ceiling effects by using recall and recognition tests in close succession and matching the young and older participants based on performance (high and low performers). The results showed detectable age differences in the recall but not in the recognition test. Therefore, the authors concluded that ceiling effects could not account for age-related differences in recognition memory.

In conclusion, the current results demonstrate that aging impairs correct recognition of pre-experimentally unfamiliar new abstract figures and non-words. Thus, it seems that the older adults have difficulties when they have to identify new items, which is comparable to previous research involving pre-experimentally familiar items (Fraundorf et al., 2019). For the shallowly encoded items, an age-related recognition impairment was found only for the abstract figures but not for the non-words. Interestingly, we did not detect overall age effects. Thus, the current study further supports the notion that recognition performance in aging is only impaired under certain conditions (Fraundorf et al., 2019) and differs for the visual and verbal domain (Umanath and Marsh, 2014; Tinard and Guillaume, 2019). Finally, these results are of particular importance, as source confusion and semantic involvement were substantially reduced by applying pre-experimentally unfamiliar items.

## Data Availability Statement

The raw data supporting the conclusions of this article will be made available by the authors, without undue reservation.

## Ethics Statement

The studies involving human participants were reviewed and approved by Ethical Committee of the Faculty of Psychology and Neuroscience of Maastricht University. The patients/participants provided their written informed consent to participate in this study.

## Author Contributions

AS, AB, and MT: conceptualization, methodology, investigation, data curation, visualization, and project administration. MT: formal analysis and writing—original draft preparation. AS and AB: resources, writing—review and editing, and supervision. All authors contributed to the article and approved the submitted version.

## Conflict of Interest

The authors declare that the research was conducted in the absence of any commercial or financial relationships that could be construed as a potential conflict of interest.

## Publisher's Note

All claims expressed in this article are solely those of the authors and do not necessarily represent those of their affiliated organizations, or those of the publisher, the editors and the reviewers. Any product that may be evaluated in this article, or claim that may be made by its manufacturer, is not guaranteed or endorsed by the publisher.

## References

[B1] Amenedo E. Diaz F. (1998). Aging-related changes in processing of non-target and target stimuli during an auditory oddball task. Biol. Psychol. 48, 235–267. 10.1016/S0301-0511(98)00040-4 9788763

[B2] Badham S. P. Maylor E. A. (2011). Age-related associative deficits are absent with nonwords. Psychol. Aging. 26, 689–694. 10.1037/a0022205 21443360

[B3] Benjamin A. S (2010). Representational explanations of “process” dissociations in recognition: the DRYAD theory of aging and memory judgments. Psychol. Rev. 117, 1055–1079. 10.1037/a0020810 20822289 PMC3045270

[B4] Benjamin A. S. Bawa S. (2004). Distractor plausibility and criterion placement in recognition. J. Mem. Lang. 51, 159–172. 10.1016/j.jml.2004.04.001

[B5] Benjamin A. S. Diaz M. Wee S. (2009). Signal detection with criterion noise: applications to recognition memory. Psychol. Rev. 116, 84–115. 10.1037/a0014351 19159149 PMC2862236

[B6] Bisiacchi P. S. Borella E. Bergamaschi S. Carretti B. Mondini S. (2008). Interplay between memory and executive functions in normal and pathological aging. J. Clin. Exp. Neuropsychol. 30, 723–733. 10.1080/13803390701689587 18608665

[B7] Bowman C. R. Dennis N. A. (2015). Age differences in the neural correlates of novelty processing: the effects of item-relatedness. Brain Res. 1612, 2–15. 10.1016/j.brainres.2014.08.006 25149192

[B8] Craik F. I (2002). Levels of processing: past, present and future? Memory. 10, 305–318. 10.1080/09658210244000135 12396643

[B9] Craik F. I. Lockhart R. S. (1972). Levels of processing: A framework for memory research. J. Verbal learn. Verbal behav. 11, 671–684. 10.1016/S0022-5371(72)80001-X

[B10] Craik F. I. Rose N. S. (2012). Memory encoding and aging: a neurocognitive perspective. Neurosci. Biobehav. Rev. 36, 1729–1739. 10.1016/j.neubiorev.2011.11.007 22155274

[B11] Craik F. I. Tulving E. (1975). Depth of processing and the retention of words in episodic memory. J. Exp. Psychol. Gen. 104, 268–294. 10.1037/0096-3445.104.3.26824156261

[B12] Czigler I. Pato L. Poszet E. Balazs L. (2006). Age and novelty: event-related potentials to visual stimuli within an auditory oddball–visual detection task. Int. J. Psychophysiol. 62, 290–299. 10.1016/j.ijpsycho.2006.05.008 16837090

[B13] Daffner K. R. Ryan K. K. Williams D. M. Budson A. E. Rentz D. M. Wolk D. A.. (2006). Increased responsiveness to novelty is associated with successful cognitive aging. J. Cog. Neurosci. 18, 1759–1773. 10.1162/jocn.2006.18.10.1759 17014379

[B14] Daffner K. R. Sun X. Tarbi E. C. Rentz D. M. Holcomb P. J. Riis J. L. (2011). Does compensatory neural activity survive old-old age? Neuroimage. 54, 427–438. 10.1016/j.neuroimage.2010.08.006 20696255 PMC2962703

[B15] Danckert S. L. Craik F. I. (2013). Does aging affect recall more than recognition memory? *Psychol. Aging. 28*, 902–909. 10.1037/a0033263 23978011

[B16] Delhaye E. Folville A. Bastin C. (2019). How to induce an age-related benefit of semantic relatedness in associative memory: it's all in the design. Psychol. Aging. 34, 572–586. 10.1037/pag0000360 31081661

[B17] Dodson C. S. Bawa S. Krueger L. E. (2007). Aging, metamemory, and high-confidence errors: a misrecollection account. Psychol. Aging. 22, 122–133. 10.1037/0882-7974.22.1.122 17385989

[B18] Fandakova Y. Lindenberger U. Shing Y. L. (2014). Deficits in process-specific prefrontal and hippocampal activations contribute to adult age differences in episodic memory interference. Cereb Cortex. 24, 1832–1844. 10.1093/cercor/bht034 23425890

[B19] Faul F. Erdfelder E. Lang A. G. Buchner A. (2007). G^*^Power 3: a flexible statistical power analysis program for the social, behavioral, and biomedical sciences. Behav. Res. Methods. 39, 175–191. 10.3758/BF03193146 17695343

[B20] Folstein M. F. Folstein S. E. McHugh P. R. (1975). Mini-mental state: a practical method for grading the cognitive state of patients for the clinician. J. Psychiatr. Res. 12, 189–198. 10.1016/0022-3956(75)90026-6 1202204

[B21] Foos P. W. Sarno A. J. (1998). Adult age differences in semantic and episodic memory. J. Genet. Psychol. 159, 297–312. 10.1080/00221329809596153 9729837

[B22] Fraundorf S. H. Hourihan K. L. Peters R. A. Benjamin A. S. (2019). Aging and recognition memory: a meta-analysis. Psychol. Bull. 145, 339–371. 10.1037/bul0000185 30640498 PMC6481640

[B23] Gallo D. A. Cotel S. C. Moore C. D. Schacter D. L. (2007). Aging can spare recollection-based retrieval monitoring: the importance of event distinctiveness. Psychol. Aging. 22, 209–213. 10.1037/0882-7974.22.1.209 17385996

[B24] Gardiner J. M (1988). Functional aspects of recollective experience. Mem. Cognit. 16, 309–313. 10.3758/BF03197041 3210971

[B25] Glass J. M (2007). Visual function and cognitive aging: differential role of contrast sensitivity in verbal versus spatial tasks. Psychol. Aging. 22, 233–238. 10.1037/0882-7974.22.2.233 17563179

[B26] Glosser G. Friedman R. B. Grugan P. K. Lee J. H. Grossman M. (1998). Lexical semantic and associative priming in Alzheimer's disease. Neuropsychology. 12, 218–224. 10.1037/0894-4105.12.2.218 9556768

[B27] Harker J. O. Riege W. H. (1985). Aging and delay effects on recognition of words and designs. J. Gerontol. 40, 601–604. 10.1093/geronj/40.5.601 4031409

[B28] Hasher L. Zacks R. May C. (1999). Inhibitory Control, Circadian Arousal and Age (Vol. XVII). Cambridge, MA: MIT Press.

[B29] Hirshman E (1995). Decision processes in recognition memory: criterion shifts and the list-strength paradigm. J. Exp. Psychol.: Learn. Mem. Cogn. 21, 302–313. 10.1037/0278-7393.21.2.302 7738502

[B30] Huang Y. Ferreira F. (2020). The application of signal detection theory to acceptability judgments. Front. Psychol. 11, 73. 10.3389/fpsyg.2020.00073 32082223 PMC7005104

[B31] Hulstijn H. J (1997). “Mnemonic methods in foreign language vocabulary learning- Theoretical considerations and pedagogical implications.” In: Second *Language Vocabulary Acquisition*, eds J. Coady and T. Huckin (Cambridge, MA: Cambridge University Press), 203–224. 10.1017/CBO9781139524643.015

[B32] Jones-Gotman M (1986). Right hippocampal excision impairs learning and recall of a list of abstract designs. Neuropsychologia. 24, 659–670. 10.1016/0028-3932(86)90005-9 3785653

[B33] Kausler D. H (1970). “Retention-forgetting as a nomological network for developmental research*,”* In: *Life-Span Developmental Psychology Research and Theory*, eds L. R. Goulet and P. B. Baltes (New York, NY: Academic Press). 10.1016/B978-0-12-293850-4.50019-4

[B34] Kim K. H. Yoon H. W. Park H. W. (2004). Spatiotemporal brain activation pattern during word/picture perception by native Koreans. Cogn. Neurosci. 15, 1099–1103. 10.1097/00001756-200405190-00003 15129153

[B35] Koutstaal W. Reddy C. Jackson E. M. Prince S. Cendan D. L. Schacter D. L. (2003). False recognition of abstract versus common objects in older and younger adults: Testing the semantic categorization account. J. Exp. Psychol.: Learn. Mem. Cogn. 29, 499–510. 10.1037/0278-7393.29.4.499 12924853

[B36] Kroll N. E. A. Knight R. T. Metcalfe J. Wolf E. S. Tulving E. (1996). Cohesion failure as a source of memory illusions. J. Mem. Lang. 35, 176–196. 10.1006/jmla.1996.0010

[B37] Malmberg K. J (2008). Recognition memory: a review of the critical findings and an integrated theory for relating them. Cogn. Psychol. 57, 335–384. 10.1016/j.cogpsych.2008.02.004 18485339

[B38] Newell B. R. Andrews S. (2004). Levels of processing effects on implicit and explicit memory tasks: using question position to investigate the lexical-processing hypothesis. Exp. Psychol. 51, 132–144. 10.1027/1618-3169.51.2.132 15114906

[B39] Noldy N. E. Stelmack R. M. Campbell K. B. (1990). Event-related potentials and recognition memory for pictures and words: the effects of intentional and incidental learning. Psychophysiology. 27, 417–428. 10.1111/j.1469-8986.1990.tb02337.x 2236443

[B40] Osborne J (2002). Notes on the use of data transformations. Pract. Assess Research and Evaluation. 8. 10.7275/4vng-5608

[B41] Overcast T. D. Murphy M. D. Smiley S. S. (1975). The effects of instructions on recall and recognition of categorized lists by the elderly. Bulletin of the Psychonomic Society. 5, 339–341. 10.3758/BF0333326723350303

[B42] Paivio A. Desrochers A. (1981). Mnemonic Techniques in Second-Language Learning. J. Educ. Psychol. 73, 780–795. 10.1037/0022-0663.73.6.780

[B43] Rajaram S (1996). Perceptual effects on remembering: recollective processes in picture recognition memory. J. Exp. Psychol.: Learn. Mem. Cogn. 22, 365–377. 10.1037/0278-7393.22.2.365 8901341

[B44] Ratcliff R (1993). Methods of dealing with reaction time outliers. Psychol. Bull. 114, 510–532. 10.1037/0033-2909.114.3.510 8272468

[B45] Redoblado M. A. Grayson S. J. Miller L. A. (2003). Lateralized-temporal-lobe-lesion effects on learning and memory: examining the contributions of stimulus novelty and presentation mode. J. Clin. Exp. Neuropsychol. 25, 36–48. 10.1076/jcen.25.1.36.13625 12607170

[B46] Ruiz Gallego-Largo T. Suengas A. G. Simon T. Pastor N. (2015). Is there a deficit in recognition in old age? Psicothema. 27, 26–31. 10.7334/psicothema2014.148 25633766

[B47] Salthouse T. A (1996). The processing-speed theory of adult age differences in cognition. Psychol. Rev. 103, 403–428. 10.1037/0033-295X.103.3.403 8759042

[B48] Seidenberg M. S. Plaut D. C. Petersen A. S. McClelland J. L. McRae K. (1994). Nonword pronunciation and models of word recognition. J. Exp. Psychol. Hum. Percept. Perform. 20, 1177–1196. 10.1037/0096-1523.20.6.1177 7844510

[B49] Smith A. D. Park D. C. Cherry K. Berkovsky K. (1990). Age differences in memory for concrete and abstract pictures. J. Gerontol. 45, 205–209. 10.1093/geronj/45.5.P205 2394917

[B50] Snodgrass J. G. Corwin J. (1988). Pragmatics of measuring recognition memory: Applications to dementia and amnesia. Human Experimental Psychology. 117, 34–50. 10.1037/0096-3445.117.1.34 2966230

[B51] Solso R. L (1995). Cognitive Psychology (4 Edn.). Boston, MA: Allyn and Bacon.

[B52] Stanislaw H. Todorow N. (1999). Calculation of signal detection theory measures. Behavior Research Methods, Instruments, and Computers. 3, 37–149. 10.3758/BF03207704 10495845

[B53] Tinard S. Guillaume F. (2019). Age-Related Differences in the Impact of Prior Knowledge on Recognition Performance: A Face Recognition Study. Exp. Aging Res. 45, 154–166. 10.1080/0361073X.2019.1586108 30870111

[B54] Titov N. Knight R. G. (1997). Adult Age Differences in Controlled and Automatic Memory Processing. Psychol. Aging. 12, 565–573. 10.1037/0882-7974.12.4.565 9416626

[B55] Toth M. Sambeth A. Blokland A. (2021a). EEG correlates of old/new discrimination performance involving abstract figures and non-words. Brain Sci. 6. 10.3390/brainsci11060719 PMC8229549 34071488

[B56] Toth M. Sambeth A. Blokland A. (2021b). The antimuscarinic agent biperiden selectively impairs recognition of abstract figures without affecting the processing of non-words. Hum. Psychopharmacol. 37, e2819. 10.1002/hup.2819 34533841 PMC9286668

[B57] Umanath S. Marsh E. J. (2014). Understanding how prior knowledge influences memory in older adults. Perspect. Psychol. Sci. 9, 408–426. 10.1177/1745691614535933 26173273

[B58] Uttl B. Henry M. Baltimore K. (2007). Are smaller age differences on old/new recognition versus free recall tests artifacts of easy memory tests?. Can. J. Exp. Psychol. 61, 374.

[B59] Verde M. F. Rotello C. M. (2007). Memory strength and the decision process in recognition memory. Mem. Cognit. 35, 254–262. 10.3758/BF03193446 17645166

[B60] Weisz J. Czigler I. (2006). Age and novelty: event-related brain potentials and autonomic activity. Psychophysiology. 43, 261–271. 10.1111/j.1469-8986.2006.00395.x 16805864

[B61] Woods D. L. Wyma J. M. Yund E. W. Herron T. J. Reed B. (2015). Factors influencing the latency of simple reaction time. Front. Hum. Neurosci. 9, 131. 10.3389/fnhum.2015.00131 25859198 PMC4374455

[B62] Yonelinas A. P (2002). The Nature of Recollection and Familiarity: A Review of 30 Years of Research. J. Mem. Lang. 46, 441–517. 10.1006/jmla.2002.286416899208

[B63] Yonelinas A. P. Kroll N. E. A. Dobbins I. Lazzara M. M. Knight R. T. (1998). Recollection and familiarity deficits in amnesia: Convergence of remember-know, process dissociation. Neuropsychology. 12, 323–339. 10.1037/0894-4105.12.3.323 9673991

